# Feasibility of 3D-Printed Brachytherapy Contrast Markers for Use With CT Planning of Interstitial Procedures

**DOI:** 10.7759/cureus.101524

**Published:** 2026-01-14

**Authors:** Paul J Black, India R Wood, Cody Lackey, Niema Razavian, Megan Lipford, Anna Snavely, Sarah Glynn, Travis Marshall, Wendy Dolesh, Mahta McKee, James Ververs, Ryan T Hughes, Bart Frizzell, Doris R Brown, Michael Farris

**Affiliations:** 1 Radiation Oncology, Atrium Health Wake Forest Baptist, Winston-Salem, USA; 2 Radiation Oncology, Novant Health, Winston-Salem, USA; 3 Radiation Oncology, Moffitt Cancer Center, Tampa, USA; 4 Radiology, Atrium Health Wake Forest Baptist, Winston-Salem, USA; 5 Biostatistics, Atrium Health Wake Forest Baptist, Winston-Salem, USA

**Keywords:** computed tomography (ct) imaging, contrast-enhanced ct, high-dose-rate interstitial brachytherapy, image-guided brachytherapy (igbt), three-dimensional (3d) printing

## Abstract

Background

Interstitial brachytherapy needles utilize radio-opaque markers that are expensive, not MR-safe, and have significant artifacts on CT/MRI. Here, we demonstrate the feasibility of 3D-printed radio-opaque markers composed of metal-infused polylactic acid (PLA) and test their use on CT-based localization of interstitial brachytherapy needles.

Methodology

Radio-opaque markers were 3D-printed using PLA filament infused with copper. Solid and hollow markers were designed, with hollow markers capable of containing viscous contrast media. Hollow markers were constructed heterogeneously with a single stripe of copper PLA along the length of a metal-free PLA to allow for feasibility testing of detecting dual contrast media using both CT and MRI. Markers were imaged via CT inside a prostate phantom and compared directly against steel and nitinol markers. Comparisons were performed in both soft tissue and air density regions. CT metal artifact (CTMA) was computed via standard deviation in the volume around each marker. Contrast-to-noise ratios (CNRs) were evaluated using CT imaging for all tested markers using identical methodology. Artifact differences between markers were compared by evaluating the standard deviations of fixed volumes around each voxel.

Results

The average CNR for solid and hollow copper PLA markers was 1.99 ± 0.07 and 1.23 ± 0.21 in tissue and 2.48 ± 0.10 and 2.99 ± 0.15 in air, respectively. The average CNR for wire and nitinol markers was 1.31 ± 0.02 and 2.33 ± 0.05 in tissue and 1.86 ± 0.01 and 2.76 ± 0.01 in air, respectively. CTMA for solid and hollow copper PLA markers were 13.62 and 8.80 HU in tissue and 9.99 and 13.66 HU in air, respectively. The average artifact for the nitinol and wire markers was 75.93 and 18.06 HU in tissue and 50.71 and 16.64 HU in air, respectively.

Conclusions

Solid copper PLA 3D-printed markers have superior visibility to commercially available steel marker wires. Both types of printed markers exhibited reduced imaging artifacts when compared with commercially available markers. Printed markers can improve the accuracy of interstitial planning and can be inexpensively produced in-house.

## Introduction

High-dose-rate (HDR) interstitial brachytherapy is a procedure in which hollow needles are inserted into a disease site to allow for the application of a therapeutic radioactive source. HDR brachytherapy can be complex, requiring as many as 40 needles to adequately treat the site. The location of each needle must be accurately defined to determine the source dwell position and calculate expected dose distributions [1-5]. MR and CT imaging are used to improve tumor and normal tissue delineation during these procedures [6]. The hollow needles utilized for interstitial brachytherapy, however, are difficult to visualize on CT or MR when empty, especially when adjacent to air. If metal stylets are in place, the needle can be visualized, but the CT metal artifact (CTMA) must be considered. To improve needle contrast and localization, commercially available stainless steel or nitinol wires are placed in each hollow needle [7]. While these wires improve delineation of needle channels, they also introduce significant CTMAs that can interfere with the mapping of needle channels and contouring anatomic structures [8]. Wires may need to be manipulated between scans, contributing to prolonged procedure times, which can introduce additional risk to the patient, as it would require that they are under anesthesia for a greater period of time.

To date, 3D printing has been used for brachytherapy applications via the creation of custom applicators, interstitial needle grids, and custom bolus [9-11]. Given the currently published data, this technology has not been applied for the creation of contrast markers to aid in the reconstruction of an interstitial implant. We hypothesize that metallic polylactic acid (PLA) infused with copper will provide a less expensive, more customizable, and MR-safe alternative to the wire markers utilized in our brachytherapy clinic. Here, we describe the design and imaging characteristics of our novel metal PLA prototypes, compare them with commercially available markers, and discuss their potential applications for use in CT-guided brachytherapy.

The goal of this study was to address the use of interstitial needles inserted without a guiding template, commonly known as free-handed needles, which is applicable to both gynecological and prostate interstitial procedures. Two 3D-printed designs were evaluated. The first was a solid cylindrical marker fabricated entirely from copper-infused PLA and intended for CT contrast visualization of interstitial needles. The second was a cylindrical, hollow prototype composed of a standard PLA with an integrated copper-infused PLA stripe. This design enables CT contrast and has the potential to permit the visualization of MR contrast encapsulated within the printed marker. CT images of all printed and commercial markers were acquired under identical conditions and assessed using contrast-to-noise ratio (CNR) and CTMA for comparison. As a feasibility test, preliminary MR images were also obtained to demonstrate the possibility of visualizing the MR contrast contained within the hollow printed marker. MR contrast optimization will be the primary focus of future development of this application. The primary objective of this study was to evaluate the suitability of these markers to serve as a CT contrast marker for interstitial brachytherapy cases. An additional exploratory objective of this study was to test the feasibility of MRI contrast visibility when inserted into hollow markers. Formal hypothesis testing was not included in this analysis as it was limited to a phantom-based, imaging feasibility study. Voxel intensity averaging and standard deviation calculations were the only statistical methods employed.

## Materials and methods

Experimental design

This work was designed as a phantom-based experimental study, evaluating the imaging performance of novel 3D-printed brachytherapy markers relative to commercially available clinical markers. Quantitative CT image quality metrics were used in comparison with an additional proof-of-concept evaluation of MR visibility. As this is a phantom study and no animal or human subjects were used, inclusion and exclusion criteria were not applicable or required. Included markers consisted of clinical stainless steel and nitinol markers, as well as custom 3D-printed markers fabricated according to the designs described in this manuscript.

Markers were designed to be compatible with standard 240 mm interstitial needles and suitable for CT imaging under clinical brachytherapy scan parameters. Markers with visible manufacturing or printing defects, incomplete copper deposition, or structural deformation were excluded from analysis. Imaging datasets with incomplete needle insertion or nonstandard scan parameters were excluded. No human subjects or patient data were included in this study.

Marker design and production

**Figure 1:**
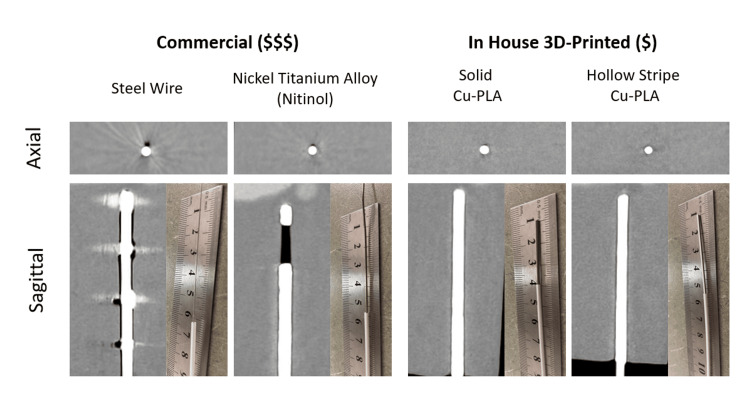
The top row demonstrates the axial view CT images of markers composed of steel wire, nitinol solid copper polylactic acid (PLA) 0.5 mm × 0.5 mm × 0.05 mm × 240 mm, and composite white Sunlu PLA+ hollow tubes 1.26 mm outer diameter, 0.8 mm inner diameter, 240 mm length, with a 0.2 mm high × 240 mm length strip of inlaid copper PLA (left to right). The bottom row represents sagittal reconstructions for each marker in the same order left to right. Inset with the sagittal planes provided are photos of each marker placed within a brachytherapy treatment needle, with a ruler provided for scale.

All 3D marker models were designed in Shapr3d for iPad. Markers were printed using a Bambu X1C printer with an attached Automatic Material System (AMS) unit, a Bambu studio slicer, and a hardened steel 0.2 mm nozzle. Models were printed on a high-temperature PEI plate. For primary comparisons to commercially available markers, two types of 3D-printed markers were created: (1) solid (0.5 × 0.5 × 240 mm) markers composed of only copper PLA and (2) composite hollow markers composed of Sunlu (Irvine, CA) white PLA with a 0.2 mm embedded stripe of copper PLA. Hollow markers were designed as a 1.26 mm tube with a flattened bottom surface for print bed adherence, and a 0.8 mm bore × 240 mm length. To test a potential application, an additional prototype marker was created with discrete contrast regions. This design can be customized to distinguish markers from one another or to define specific source dwell positions. Figure 1 shows an axial and sagittal cross-section CT image of this prototype marker, along with a photo of each marker for scale.

Marker imaging and statistical analysis

CT images were acquired using a GE Healthcare (Chicago, IL) Wide Bore CT simulator in helical scanning mode. Standard scan settings for a clinical brachytherapy case (slice thickness of 2.5 mm, 120 kV, and 100 mA) were used for all scans. To demonstrate the feasibility of MR contrast enhancement, hollow markers were filled with a glycerin-based gel. Images were acquired using a Siemens (Washington, D.C.) Vida 3.0 T MR simulator and a Siemens Avanto fit 1.5 T MR scanner. Specific pulse sequences used were T1 sagittal MPRAGE and T2 sagittal 2D BLADE fast spin echo with fat saturation. Identical T1 and T2 pulse sequences were used for both 1.5 T and 3.0 T MR scanners. MR imaging of these markers will be the focus of future work, but preliminary images demonstrate the feasibility of dual modality contrast.

To best simulate an interstitial implant scenario, 240 mm interstitial needles (Elekta, Atlanta, GA) were inserted into a prostate ultrasound phantom (CIRS Model 053S, Norfolk, VA), which is safe for both CT and MR imaging. Needles were left in place for all scans to best compare dissimilar materials. All image analyses were performed using ImageJ, MIM, and RayStation software packages.

**Figure 2:**
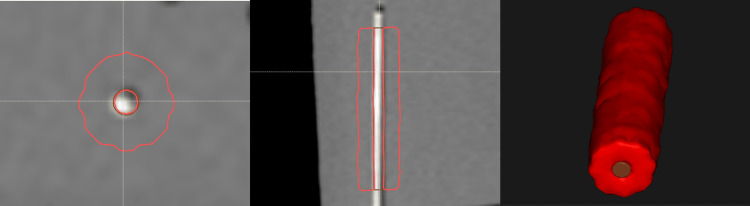
Contours used in analysis of CT images. For each channel, the needle was contoured in addition to a rind around the needle with a wall thickness of 5 mm. The rind region was used to quantify the metal artifact, while both volumes were used to calculate contrast-to-noise ratios.

Statistical analyses were performed to evaluate CNR and CTMA for each marker type using the mean and standard deviation. To quantify CTMA, a contour was created around each needle within the phantom that included the marker. Specifically, a fixed cylindrical contour was created with dimensions of 1 mm radius and 20 mm length to encapsulate each marker. Contour algebra was then performed to create a contour that encapsulated the volume around the marker. This volume was a cylindrical shell that is the result of removing a 4 mm diameter, 20 mm length cylindrical volume from a 24 mm diameter, 100 mm length cylinder. Using these fixed geometry rules mitigates the possibility of inter-observer or intra-observer variability. On CT imaging, a standard deviation was calculated in a region of 1 cm around each marker. Figure 2 provides a visual example of the volumes used in CT image analysis. For CT imaging, a CNR was calculated using a homogeneous region of tissue equivalent material within 2 mm of the marker. The following Equation 1 [12] was used to estimate the CNR for each marker:

$C=\frac{|S_{A}-S_{B}|}{\sigma_{0}}$

where S_A_ is the average signal of the volume encapsulating the marker of interest, S_B_ is the signal from a homogeneous tissue region, and $\sigma_{0}$ is the standard deviation of a region encompassing a volume of 2 mm around each marker.

Raw voxel values for all relevant contours were imported into Microsoft Excel for analysis. For metal artifact evaluation, standard deviations were compared between the clinical standard markers (wire and nitinol) and the 3D-printed markers (Solid Copper PLA and Copper PLA Stripe). The same approach was used for the CNR analysis, with the standard deviation of CNR values calculated from three images of three distinct markers for each marker type used to estimate CNR uncertainty. No formal hypothesis testing was performed, as the primary objective of this phantom-based study was comparative performance evaluation and feasibility assessment rather than inferential statistical testing. Differences between marker types were interpreted based on the relative magnitude and consistency of observed metrics.

## Results

CT imaging

CT images of markers fabricated using the 3D-printing technique, as well as the commercially available markers, are shown in Figure 1. Compared to the commercially available markers, each 3D-printed marker demonstrated a reduced CTMA. This improvement in CTMA was similar for both solid and hollow printed markers in both tissue- and air-equivalent media. For comparison with commercial markers, we focused exclusively on copper PLA as opposed to brass and bronze PLA, as this material was associated with fewer nozzle clogs and printing inconsistencies during prototype development.

**Figure 3:**
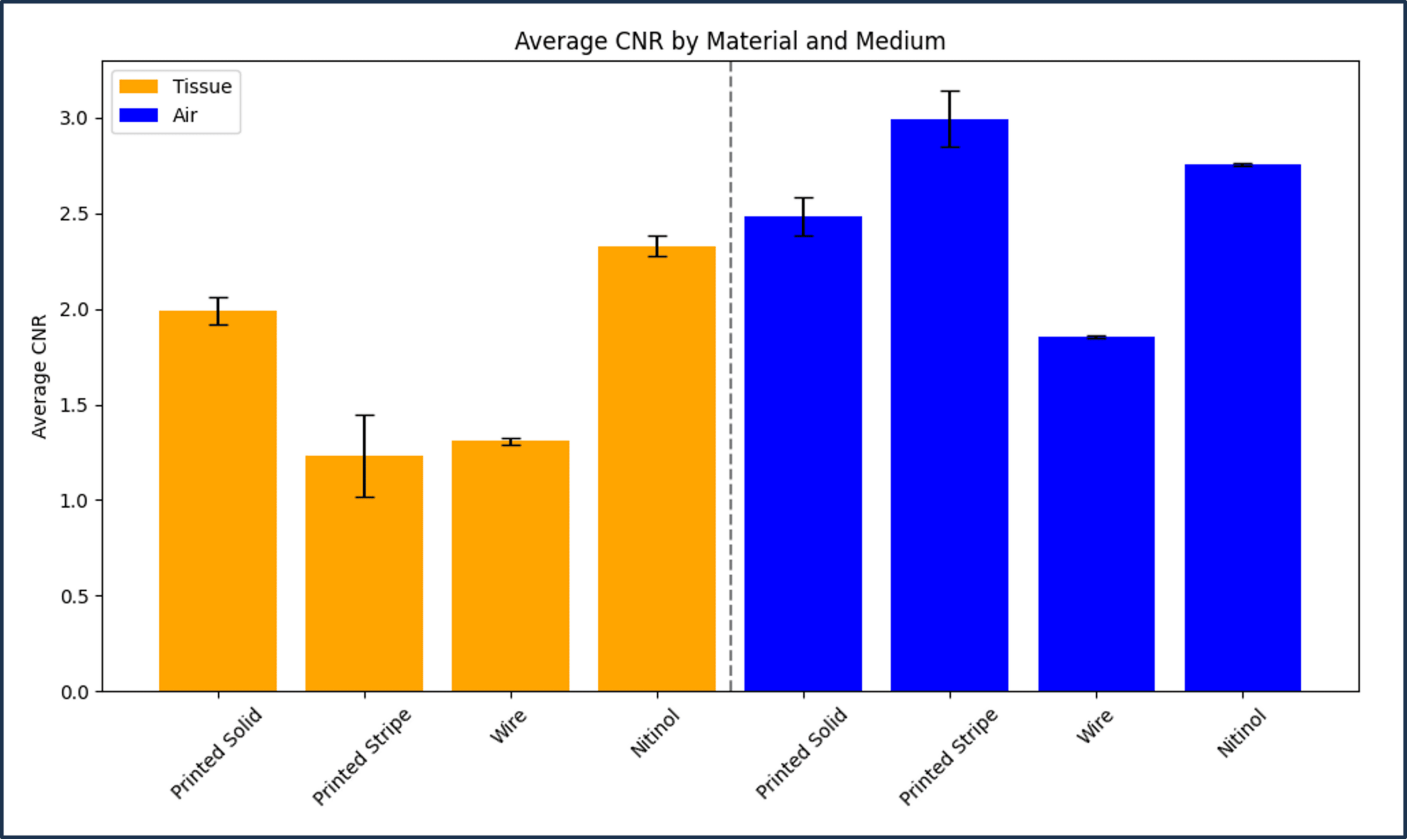
Histogram of contrast-to-noise ratio (CNR) of all tested markers imaged using CT. CNR was quantified in both tissue-equivalent and air media. Provided error bars were calculated using the CNR standard deviation across three CT images of three different markers for each type analyzed.

**Figure 4:**
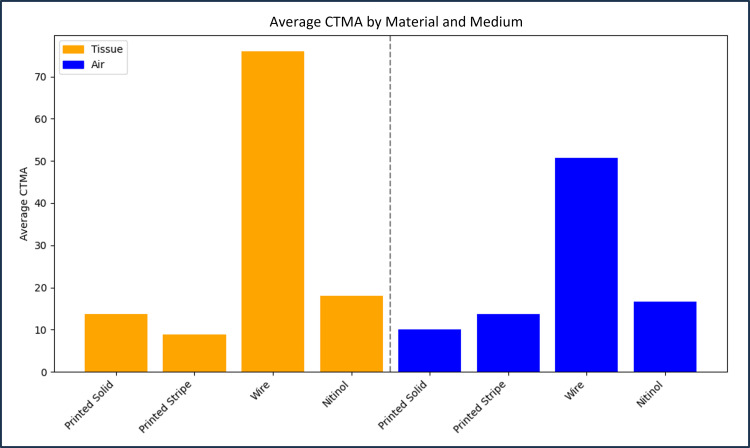
Histogram of the CT metal artifact (CTMA) of all tested markers imaged using CT. CTMA was calculated via the standard deviation of the rind region around each marker. For each marker, the reported value is an average of three markers for each type, imaged separately.

**Figure 5:**
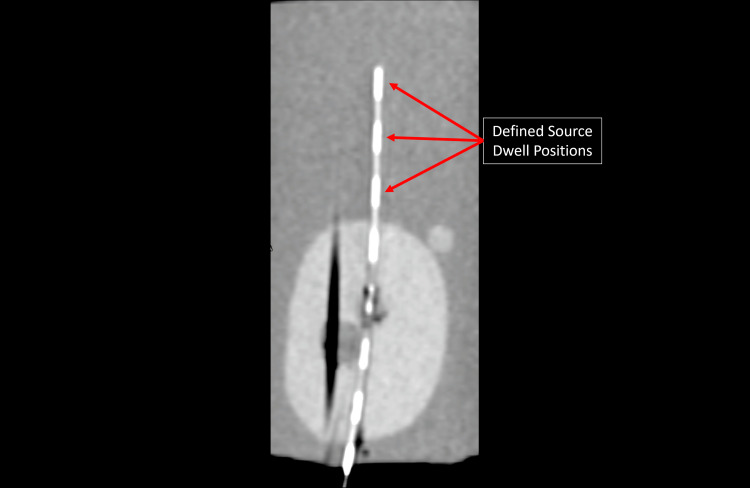
Sagittal plane of a CT image of a prototype marker designed to have discrete contrast regions. Each region is 1 cm long with a spacing of 1 cm between each region. The length of the contrast regions and the spacing between them is customizable and readily observable on CT images.

Figure 3 and Figure 4 provide CNR and CTMA histogram results for all tested markers imaged with CT. Table 1 and Table 2 summarize the values provided in these figures for each type of marker. The average CNR for solid and hollow copper PLA markers was 1.99 ± 0.07 and 1.23 ± 0.21 in tissue and 2.48 ± 0.10 and 2.99 ± 0.15 in air, respectively. The average CNR for wire and nitinol markers was 1.31 ± 0.02 and 2.33 ± 0.05 in tissue and 1.86 ± 0.01 and 2.76 ± 0.01 in air, respectively. CTMA for solid and hollow copper PLA markers were 13.62 and 8.80 HU in tissue and 9.99 and 13.66 HU in air, respectively. The average artifact for the nitinol and wire markers was 75.93 and 18.06 HU in tissue and 50.71 and 16.64 HU in air, respectively. Hollow copper PLA markers exhibited significantly lower CNR than nitinol and showed no significant difference when compared with steel wire (Figure 3, Table 1). Finally, the prototype marker with defined contrast regions was easily visualized on CT images, with an example of a sagittal plane of this marker provided in Figure 5.

**Table 1:** Measurement of contrast-to-noise ratio (CNR) in both tissue-equivalent and air regions. CNR values were calculated using Equation 1. Provided errors were calculated using a standard deviation of CNR for three of each marker type each imaged individually.

Medium	Material	Average CNR
Tissue	Printed solid	1.99 ± 0.07
	Printed stripe	1.23 ± 0.21
	Wire	1.31 ± 0.02
	Nitinol	2.33 ± 0.05
Air	Printed solid	2.48 ± 0.10
	Printed stripe	2.99 ± 0.15
	Wire	1.86 ± 0.01
	Nitinol	2.76 ± 0.01

**Table 2:** Average CT metal artifact (CTMA) for each marker in both tissue-equivalent and air regions. CTMA was calculated using the standard deviation of a defined voxel volume around the marker of interest.

Medium	Material	Average CTMA
Tissue	Printed solid	13.62
	Printed stripe	8.8
	Wire	75.93
	Nitinol	18.06
Air	Printed solid	9.99
	Printed stripe	13.66
	Wire	50.71
	Nitinol	16.64

MR imaging feasibility for hollow marker

**Figure 6:**
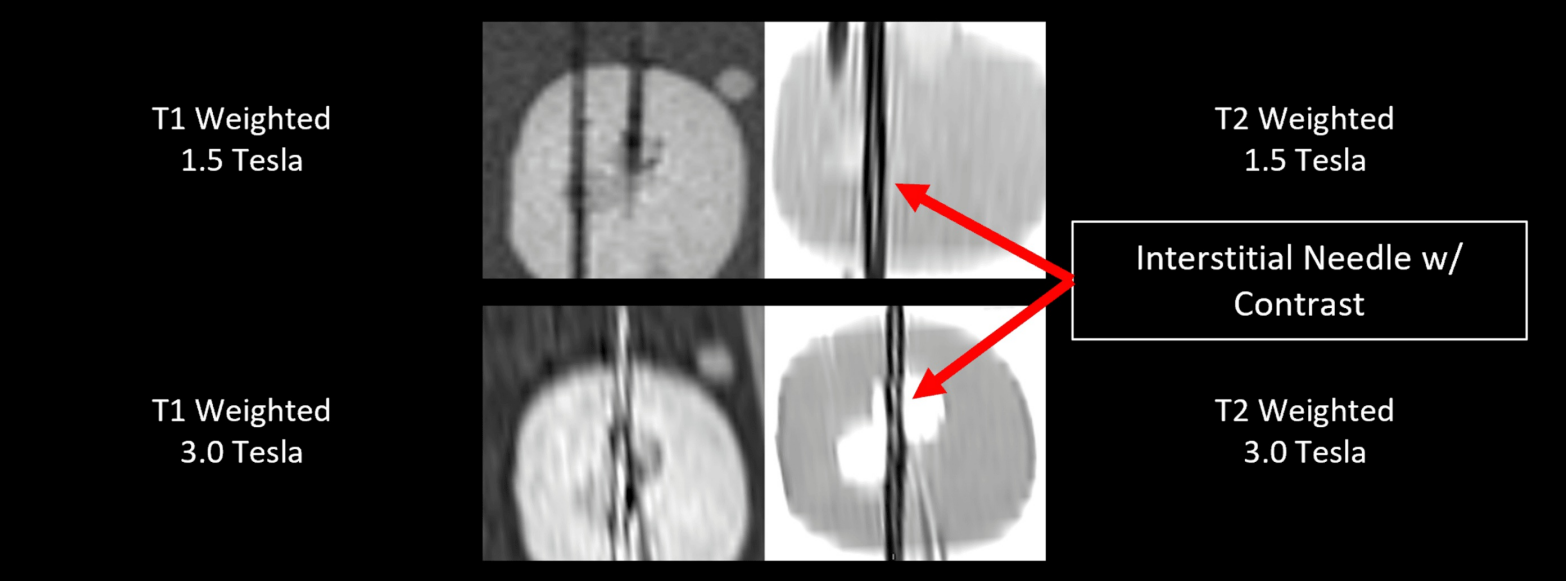
Sagittal slices of MR images taken at different B0-field strengths and pulse sequences. Top left: 1.5 T, T1-weighted; top right: 1.5 T, T2-weighted; bottom left: 3.0 T, T1-weighted; bottom right: 3.0 T, T2-weighted. Markers were visible on all scans except for 1.5 T, T1-weighted.

Composite markers filled with glycerin gel were visible on both 1.5 T and 3.0 T clinical MR scanners, but refinement will be required to achieve clinically useful contrast. Visibility was maintained at both field strengths for T2-weighted pulse sequences, whereas T1-weighted sequences yielded favorable results only at 3.0 T magnetic field strength. Figure 6 provides initial MR images of prototype markers for multiple pulse sequences and B-field strengths. Contrast is visible in three of the four imaging sequences, but further development will be required before this can be attempted on live patients. All MR images were obtained at the highest spatial resolution available for each employed pulse sequence.

## Discussion

This work presents a novel and innovative application of 3D printing in radiation oncology. Based on the available literature, this is the first use of metallic PLA filaments as CT and MR contrasted markers and the first brachytherapy marker intended for visualization in both MR and CT imaging modalities.

Table 1 outlines the CNR for all marker types in both tissue-equivalent and air regions. Solid copper PLA markers exhibited the highest CNR in tissue, while hollow copper PLA markers showed lower CNR in tissue but comparable or higher CNR in air relative to wire and nitinol markers. Nitinol markers had higher CNR than wire markers in tissue but not in air, showing that performance differences are dependent on the imaging environment and marker composition. As summarized in Table 2, both solid and hollow copper PLA markers exhibited lower CTMA than commercially available stainless steel wire and nitinol markers in both tissue-equivalent and air regions. The enhanced CTMA performance of the printed-stripe prototype in both tested media, compared with commercial markers, provides a distinct advantage over standard contrast markers used in brachytherapy, particularly in cases where needles are positioned in close proximity. In such cases, CTMA can introduce added difficulties in localizing treatment channels on CT images in clinics lacking CT units with metal artifact reduction (MAR). Although empty needles are sometimes used for imaging, providing sufficient contrast in tissue without metal-induced artifacts, this approach is problematic when needles are located within air pockets, where there is little to no discernible contrast between the empty needle and the surrounding air. For this reason, image analysis was performed in both open air and tissue-equivalent regions.

In addition, the shape and composition of the printed markers are customizable with user-friendly tools, making this technique more versatile than commercially available, fixed-geometry markers. This capability is demonstrated by the prototype marker shown in Figure 5, in which CT contrast regions were printed at 1 cm increments with 1 cm gaps between each contrast region. The lengths of both the gaps and the contrast regions can be easily adjusted, enabling the creation of distinct markers for channel identification on CT imaging while maintaining the advantage of low CTMA. Although stainless steel wire markers are designed with distinct contrast patterns, they introduce substantially greater CTMA than the fabricated markers presented in this study.

Production speed is another major advantage of this technique. A set of 24 composite markers was printed in less than two hours, while a set of solid markers was printed in approximately 30 minutes. This avoids delays in ordering and shipping markers. If necessary, markers could be printed on the day of the procedure.

The required materials, less than 1 g of filament per marker, result in a device costing about two cents apiece. PLA can be sterilized [13] and reused. Additionally, the 3D printer is inexpensive, with prices ranging from $1,000 to $2,500, representing a one-time investment that is far lower than standard clinical equipment [14]. Given the modest upfront cost and low material expense, this process is both sustainable and cost-effective. By varying the material composition and placing material at well-defined intervals along the marker’s length, markers can be created for any brachytherapy applicator or needle size, allowing clinicians to better define dwell positions. It is important to note that hollow markers require non-continuous metal-infused PLA sections if MR image contrast is sought. Early prototypes featuring hollow copper PLA markers effectively shielded the MR contrast material from RF pulses, eliminating any potential visualization on MR images. Investigations into the clinical utility of CT contrast customization and MR contrast of these markers in interstitial brachytherapy will be the focus of future work.

Limitations

The findings of this study should be interpreted within the context of its defined experimental scope. All evaluations were performed in a controlled phantom environment, which enables reproducible comparison of imaging performance but does not capture the full complexity encountered during clinical interstitial brachytherapy procedures, including tissue heterogeneity, patient motion, and anatomical variability. MAR was not an available algorithm on our imaging units, so it was not used in this study. While inter-observer and intra-observer variability were not formally evaluated, we chose a methodology with fixed parameters to mitigate its influence on the results presented here.

This work was intentionally focused on CT-based image quality metrics, with MR imaging included as an initial feasibility assessment. Comprehensive optimization of MR contrast behavior and detailed characterization across a broader range of pulse sequences were beyond the scope of this investigation and are planned for future studies.

Finally, sterility, mechanical robustness, dosimetric impact, and clinical workflow integration were not formally evaluated. Only a limited number of marker designs and material compositions were studied, and performance may vary with alternative geometries, filament formulations, or printer configurations. Although PLA materials can be sterilized and the markers were designed to be compatible with standard interstitial needles, clinical implementation would require additional quality control validation, including mechanical testing and testing under clinical use conditions.

## Conclusions

The 3D-printed markers developed in this study are inexpensive and demonstrate improved imaging performance compared with standard brachytherapy markers, exhibiting higher CNR, lower CTMA, and visibility on both CT and MR imaging. Unlike commercially available markers, which are expensive and not MR-safe, these markers have the potential to improve the dwell position identification and the accuracy of interstitial brachytherapy implants, supporting more precise treatment planning, including MR-only workflows. The flexibility to vary material composition and structure enables customization for different applicators and needle sizes, allowing versatile clinical use across a range of scenarios. Combined with the low cost of the materials and the modest one-time investment in a 3D printer, this approach is both accessible and sustainable for routine clinical practice. Future work will focus on (1) evaluating the clinical utility of these markers in patient procedures, (2) optimizing their design for broader adoption in interstitial brachytherapy, and (3) expanding on the feasibility data presented in this manuscript through an MRI-focused study.
